# Validation and reliability of the rapid diagnostic test ‘SD Bioeasy Dengue Duo’ for dengue diagnosis in Brazil: a phase III study

**DOI:** 10.1590/0074-02760170433

**Published:** 2018-06-25

**Authors:** Paulo Sousa Prado, José Teófilo Duarte Almeida, Lanna Takada de Abreu, Cristina Gabriel Silva, Larissa da Costa Souza, Marizoneide Cavalcante Gomes, Lucinda Malheiros Teixeira Mendes, Eliane Maria dos Santos, Gustavo Adolfo Sierra Romero

**Affiliations:** 1 Universidade de Brasília, Núcleo de Medicina Tropical, Brasília, DF, Brasil; 2 Laboratório Central de Saúde Pública, Brasília, DF, Brasil

**Keywords:** dengue, dengue diagnosis, immunochromatographic rapid tests, accuracy

## Abstract

**BACKGROUND:**

The diagnosis of dengue is complex. Until recently, only specialised laboratories were able to confirm dengue infection. However, this has changed with the newly available immunochromatographic rapid tests. Early diagnosis is of great interest, and point-of-care rapid tests have been increasingly used in Brazil. Most of those tests have not undergone validation in the Brazilian population. In this context, we decided to evaluate a rapid test introduced in the Federal District (FD).

**OBJECTIVES:**

To estimate the accuracy and reliability of the SD Bioeasy Dengue Duo rapid test and its components to detect dengue infections in a consecutive sample of symptomatic residents in the FD, Brazil.

**METHODS:**

In total, 1353 venous blood samples were collected between 2013 and 2014. Two hundred and six positive samples (cases) and 246 negative samples (non cases) were required for sensitivity and specificity estimation, respectively; for agreement evaluation, we used 401 samples. The reference standard used was a composite of MAC-ELISA, virus isolation and real-time polymerase chain reaction (RT-qPCR). The evaluation was conducted prospectively under field conditions in the public health units of the FD.

**FINDINGS:**

The results for the overall accuracy of the rapid test (NS1/IgM combined) showed 76% sensitivity and 98% specificity. The sensitivity for the NS1 component (67%) was better than that for the IgM component (35%). The positive likelihood ratio was 46, and the negative likelihood ratio was 0.24. The reliability of the test (NS1/IgM combined) demonstrated crude agreement of 98% (Kappa index 0.94).

**MAIN CONCLUSIONS:**

The present phase III, large-scale validation study demonstrates that the rapid test SD Bioeasy Dengue Duo has moderate sensitivity (NS1/IgM combined) and high specificity. Therefore, the test is useful in confirming the diagnosis of dengue, but not enough to rule out the diagnosis. Our results also suggest that Dengue virus (DENV) viral load estimated through the RT-qPCR and antibody level measured through the MAC-ELISA could have had a direct influence on the accuracy of the rapid test.

Dengue is the most disseminated arthropod-borne viral disease among humans. The World Health Organization (WHO) estimates that 50 million people are infected yearly in the world and approximately 2.5 billion live in areas with high-risk of infection. The number of cases has increased approximately 30-fold for the past 50 years, and the reason is likely related to human population increases and urbanisation (WHO 2009). These numbers are probably underestimated, as other authors using cartographic approaches estimate approximately 390 million dengue infections per year, of which 96 million would be apparent (Bhatt et al. 2013). Cities create larvae developing natural reservoirs for *Aedes aegypti*, the main vector for Dengue virus (DENV) (Wang and Sekaran 2010, Gubler 2011). There are four distinct viral serotypes that cause dengue (DENV-1, DENV-2, DENV-3 and DENV-4). The four serotypes can cause infection in humans and are globally distributed (Messina et al. 2014) causing either asymptomatic or symptomatic infections, some of them with lethal outcomes (Paixão et al. 2015, Tassara et al. 2017).

The new WHO classification for dengue severity is divided into Dengue without Warning Signs, Dengue with Warning Signs, and Severe Dengue to better and more precisely define the progression of the disease and set up proper public policies (Horstick et al. 2015). In Brazil, the Health Ministry advises insect vector control to reduce virus transmission and early case detection to reduce the lethality rate in severe cases (Cavalcanti et al. 2013). Current dengue treatment is based on isotonic fluid replacement since no specific antiviral intervention is available (Guzmán and Kourí 2004). There is a registered vaccine in Brazil with low efficacy and no effect evaluation in children and old-aged people (Shim 2017). Consequently, the accurate and reliable diagnosis is of great interest for clinical management and avoiding disease progression towards severe manifestations (Guzmán et al. 2010).

Dengue diagnosis remains a major challenge for most Latin American countries. The laboratory confirmation of the diagnosis is complex and, until recently, only specialised laboratories were able to confirm the infection. However, this scenario has changed gradually with the availability of immunochromatographic rapid tests (RDTs), which detect dengue infection quickly. RDTs can be applied in the lab or as point-of-care tools. Among several techniques available for dengue diagnosis, the immunochromatographic rapid tests have been increasingly used in Brazil. Nonetheless, most of the available tests have not undergone extensive validation in the Brazilian population. Additionally, precise accuracy data are not currently available for clinicians acting in routine clinical settings (Osorio et al. 2015). Therefore, this research aims to estimate the accuracy and reliability of the SD Bioeasy Dengue Duo (Standard Diagnostic Inc., Korea) and its components to detect acute dengue infection in a consecutive sample of symptomatic residents in the Federal District (FD), Brazil.

## MATERIALS AND METHODS

*Study design* - The study was divided into two components: (1) a large-scale phase III validation carried out in a consecutive sample of dengue-suspicious patients from the target population (Schilling et al. 2004); and (2) a reliability study among the different public units where samples were collected. The samples were separated into acute phase (up to within seven days of onset of symptoms) and convalescent phase (more than seven days since onset of symptoms).

*Study site* - The Brazilian FD is divided into 31 regions. We collected samples from six public units: five hospitals located in different regions (Ceilândia, Guará, Taguatinga, Sobradinho, and Planaltina) and the reference Central Public Health Laboratory of the FD (Portuguese acronym, LACEN-DF), for diagnosis of infectious diseases in the FD.

*Suspected dengue case definition and recruitment* - Potential participants with dengue symptoms that spontaneously sought treatment in the selected healthcare units between July 2013 and July 2014 were consecutively enrolled in the study. A suspected dengue case was defined as a patient with fever lasting up to seven days, accompanied by at least two of the following symptoms: headache, retro-orbital pain, myalgia, arthralgia, prostration, rash and exposure to dengue transmission area in the last 15 days.

*Sample size and reference standard* - Sample size was calculated with the following assumptions: α = 0.05, desired precision = +4%, expected sensitivity = 92.9%, and expected specificity = 88.8%. Then, 160 positive samples (cases) and 240 negative samples (non-cases), as classified by the reference standard, would be required for sensitivity and specificity estimation, respectively. The reference standard used was a composite of MAC-ELISA, virus isolation, and real-time polymerase chain reaction (RT-qPCR). Samples were considered as true-positives (cases) when at least one of the reference tests had a positive result, and true-negatives (non-cases) when all the three reference tests were negative. All the reference tests were carried out in LACEN-DF.

*Index test* - The rapid test Bioeasy SD Dengue Duo is a qualitative immunoassay for simultaneous detection of NS1 antigen, IgM antibodies and IgG antibodies for dengue in serum, plasma or whole blood. All samples were tested with the index test at the health units where the participant was recruited, following the instructions of the manufacturer.

*Masking* - Reference tests and the index test were performed independently and masked. Professionals who performed reference tests did not know the result of the index test and vice versa.

*Reliability evaluation* - The reliability between the results obtained in the health units and the reference laboratory was evaluated in 401 samples and measured by the percentage of crude agreement and the kappa coefficient (κ) with the respective 95% confidence intervals (95% CI) (Sim and Wright 2005).

*Laboratory methods* - For RT-qPCR, viral RNA from serum samples, stored in a −70°C freezer, was extracted with the High Pure Viral Nucleic Acid Version 18 Kit (Roche Diagnostics). The reaction was performed in a single step (multiplex), following the kit protocol, and using LightCycler ® Multiplex RNA Virus Master Version 03 (Roche Diagnostics). The final volume was 20 μL, the primers' concentrations were 0.5 μM, and probe concentration was 200 nM. For the interpretation of the results, the threshold cycle (Ct) of each reaction was taken into account. For Cts between 1 and 37, the sample was considered positive; otherwise, specimens were considered negative (Johnson et al. 2005).

The technique used for viral isolation from the sera is based on the sensitivity of *Aedes albopictus* cells (C6/36 clone) to several flaviviruses. From each serum sample, 20 μL was inoculated into culture tubes containing C6/36 cells, which were incubated at 25°C for approximately eight days. Confirmation of viral infection was obtained by indirect immunofluorescence with monoclonal antibodies for DENV 1, DENV 2, DENV 3, DENV 4 and Yellow Fever (Gubler et al. 1984).

Serology testing was carried out using MAC-ELISA, according to the protocol by Kuno et al. (1987). The reading test was done by colorimetric methods using a spectrophotometer for ELISA plates (ASYS Expert Plus) with a 405 nm filter. The cut-off point in absorbance was 0.2.

For the implementation of the Bioeasy SD Dengue Duo rapid test (Standard Diagnostic Inc., Korea), all the manufacturer's recommendations were followed. Visual interpretation of test results was done 15 to 20 minutes after sample addition. Samples were considered positive for the rapid test when a positive result was obtained for the NS1 and/or IgM bands.

*Ethics* - The Ethics Committee of the Health Secretariat of the Federal District approved this project under the register number 20472313.3.0000.5553. Free and informed consent was obtained from all participants included in the research.

## RESULTS

**Fig. 1:**
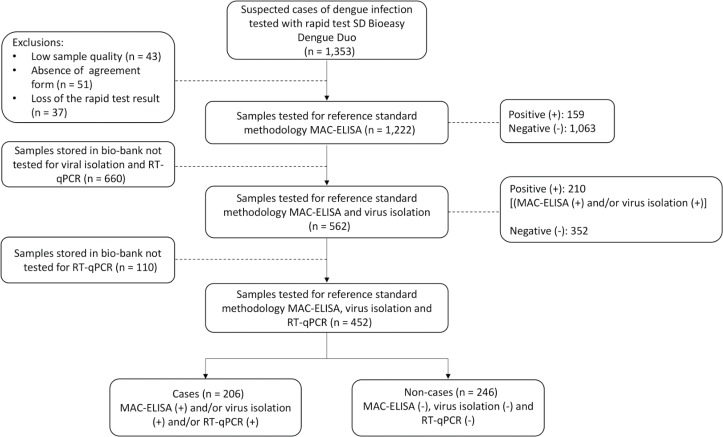
flow diagram for the study of the accuracy of the SD Bioeasy Dengue Duo rapid test. Federal District (FD), Brazil, 2014.

Of the 1,353 participants who met the suspected dengue case definition and were tested with the index test, 1,222 were consecutively tested by the reference standard methodologies, resulting in the identification of 206 cases and 246 non-cases (Fig. 1).

The characteristics of the participants are shown in Table I. The reference tests among cases (n = 206) showed positive in 143 samples for the MAC-ELISA, 58 for viral isolation and 121 for RT-qPCR (Table II). DENV- 1, DENV-3 and DENV-4 were detected by RT-qPCR, with the highest frequency for serotype 1 in 95.04% of the samples. Cases and non-cases were similar with respect to demographic and clinical characteristics. Most cases and non-cases had no history of previous episodes of dengue at baseline. Regarding the time from onset of the suspected dengue syndrome, participants were classified into acute (≤ 7 days) or convalescent (> 7 days). Most cases and non-cases were in the acute phase, 81% and 79%, respectively (Table III).

**TABLE I:** Demographic and clinical characteristics of 1,222 participants from Federal District (FD) with suspected dengue syndrome in 2014

Characteristics		Values (% or SD)
Sex (%)		
	Male	499 (40.83)
	Female	723 (59.16)
Age (years)		
	Mean (SD)	31.44 (± 18.12)
	Variation	1 - 92
	Median	36
Duration of symptoms (%)		
	≤ 5 days	841 (68.82)
	> 5 days	273 (22.34)
	Not informed	108 (8.83)
	Mean (SD)	4.60 (± 4.81)
	Median	2.5
History of previous dengue episode (%)		
	Yes	101 (8.26)
	No	962 (78.72)
	Not informed	159 (13.01)
Public health unit (%)		
	Sobradinho	61 (4.99)
	Planaltina	1106 (90.50)
	Guará	1 (< 1)
	LACEN	1(< 1)
	Taguatinga	33 (2.70)
	Ceilândia	20 (1.63)

SD: standard deviation.

**TABLE II:** Result composition for 206 cases regarding three standard methodologies of reference: MAC-ELISA IgM, virus isolation and real-time polymerase chain reaction (RT-qPCR). Federal District (FD), 2014

Reference standard (composition)	n (%)
Mac IgM + / Isolation - / qPCR -	84 (40.77)
Mac IgM + / Isolation - / qPCR +	50 (24.27)
Mac IgM - / Isolation + / qPCR +	48 (23.30)
Mac IgM - / Isolation - / qPCR +	14 (6.79)
Mac IgM + / Isolation + / qPCR +	9 (4.36)
Mac IgM - / Isolation + / qPCR -	1 (0.005)
Total	206 (100)

**TABLE III:** Clinical and demographic characteristics of cases and no cases for the validation and reliability of the rapid test ‘SD Bioeasy Dengue Duo’, Federal District (FD) 2014

Variables		Cases n = 206 Values (%)	No cases n = 246 Values (%)
Sex			
	Male	84 (40.78)	90 (36.59)
	Female	122 (59.22)	156 (63.41)
Age			
	0 - 20	47 (22.82)	84 (34.14)
	21 - 40	79 (38.35)	102 (41.47)
	41 - 60	60 (29.12)	42 (17.07)
	61 - 80	19 (9.22)	14 (5.70)
	81 - 100	1 (0.49)	4 (1.62)
	Mean (SD)	35.83 (SD = 17.63)	31.19 (SD = 18.23)
	Median	29.5	36
Duration of symptoms (days)			
	≤ 2	42 (20.39)	75 (30.49)
	3 - 4	55 (26.69)	77 (31.30)
	5 - 6	53 (25.73)	34 (13.83)
	≥ 7	43 (20.88)	30 (12.19)
	Not informed	13 (6.31)	30 (12.19)
Public health unit			
	Sobradinho	9 (4.36)	0
	Planaltina	187 (90.78)	207 (84.15)
	Guará	0	1 (0.40)
	LACEN	0	1 (0.40)
	Taguatinga	6 (2.92)	22 (8.96)
	Ceilândia	4 (1.94)	15 (6.09)
History of previous dengue episode (%)			
	Yes	21 (10.19)	19 (7.73)
	No	148 (71.84)	188 (76.42)
	Not informed	37 (17.97)	39 (15.85)
Infection state			
	Acute phase	167 (81.06)	195 (79.27)
	Convalescent phase	26 (12.63)	21 (8.54)
	Not informed	13 (6.31)	30 (12.19)

SD: standard deviation.

Of the 246 true-negative samples, four showed a false-positive result to the rapid test Bioeasy SD Dengue Duo. Two were positive for NS1 and negative for IgM, and two were negative for NS1 and positive for IgM. Among the 206 cases, 49 samples had false negative results to the rapid test. The test accuracy parameters are described in Table IV.

**TABLE IV:** Reliability of the rapid test ‘SD Bioeasy Dengue Duo’, Federal District (FD), 2014

Index test	Sensitivity (%) (n = 206) [CI 95%]	Specificity (%) (n = 246) [CI 95%]	PPV (%) [CI 95%]	NPV (%) [CI 95%]	PLR [CI 95%]	NLR [CI 95%]
SD	76.21	98.37	97.51	83.16	46	0.24
Bioeasy Dengue Duo	(157/206)	(242/246)	(157/161)	(242/291)	(0.7621/0.0163)	(0.24/0.98)
NS1/IgM	[70.3 to 81.6]	[96.2 to 99.7]	[95.4 to 98.9]	[79 to 86.9]	[14.3 to 100.6]	[0.09 to 0.63]
SD	67.96	99.18	98.58	78.70	82	0.33
Bioeasy Dengue Duo	(140/206)	(244/246)	(140/142)	(244/310)	(0.6756/0.0082)	(0.33/0.99)
NS1	[61.1 to 72.8]	[97.8 to 100]	[96 to 99.9]	[74 to 81.9]	[25.2 to 177.4]	[0.12 to 1.14]
SD	35.92	99.18	97.36	64.89	43	0.65
Bioeasy Dengue Duo	(74/206)	(244/246)	(74/76)	(244/376)	(0.3552/0.0082)	(0.65/0.99)
IgM	[29.1 to 40.8]	[97.8 to 100]	[95 to 98.9]	[60 to 67.9]	[13.2 to 92.7]	[0.24 to 1.72]

PPV: positive predictive value; NPV: negative predictive value; PLR: positive likelihood ratio; NLR: negative likelihood ratio.

The results of the reference tests in the 49 participants with false-negative results observed with the rapid test are demonstrated in Supplementary data (Table I). Thirty-eight of the 49 (77,6%) samples with false-negative results were positive for the MAC-ELISA and 15 (30,6%) were positive for RT-qPCR. These results indicate that for those 49 false negative samples, 67.34% (33 samples) had a combination of MAC-ELISA positive, viral isolation negative and RT-qPCR negative reference results.

The analysis of the performance of each component of the rapid test demonstrated that, among 143 positive samples for serum IgM MAC-ELISA, only 72 (50.34%) were positive for the IgM component of the rapid test, while 90 (62.93%) positive specimens were observed for the NS1 component [Supplementary data (Table II)].

We also analysed the performance of the rapid test in different scenarios of dengue infection: acute infection, convalescence and participants who reported a history of a previous episode of dengue [Supplementary data (Tables III-IV)].

The rapid test showed 78.44% (131/167) sensitivity for acute infection when we analyse the NS1 and IgM components together [Supplementary data (Table III)]. For reinfection analysis, 148 samples were classified as primary dengue and 21 as secondary dengue. The sensitivity of the rapid test proved to be better in primary dengue (77.7%) than in secondary dengue (66.7%), independent of whether the rapid test components (NS1/IgM/IgG) were being analysed separately or together [Supplementary data (Table IV)].

**Fig. 2:**
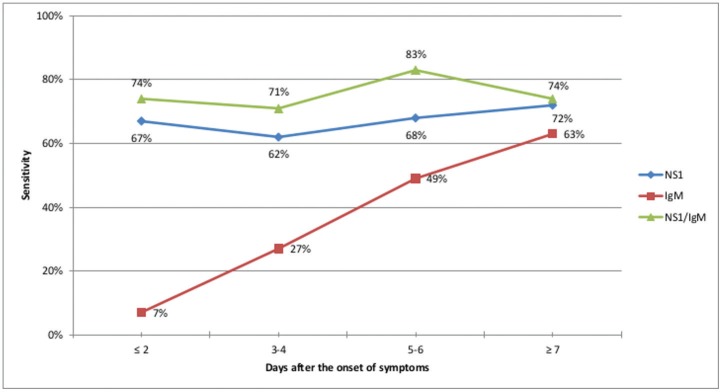
sensitivity evaluation of the NS1 component, IgM component and the combination of both NS1 and IgM components of the SD Bioeasy Dengue Duo test according to the time from onset of symptoms to collection of the serum sample. Federal District (FD), Brazil, 2014.

The sensitivity of both the IgM component and the NS1 component gradually improved as the number of days from onset of symptoms to the sample collection day increased (Fig. 2).

**Fig. 3:**
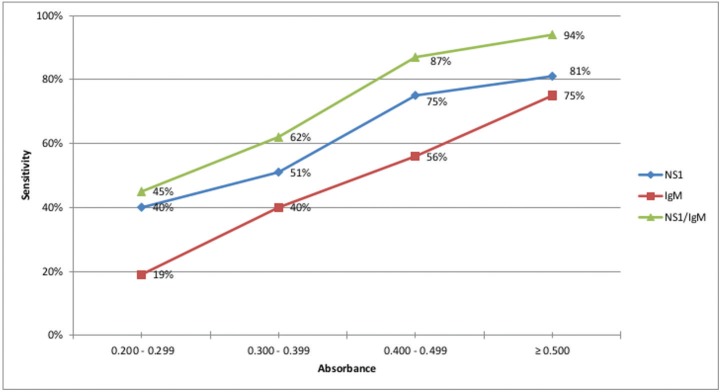
sensitivity evaluation of the NS1 component, IgM component and the combination of both NS1 and IgM components of the SD Bioeasy Dengue Duo test according to the absorbance intensity of the reference standard test MAC-ELISA-IgM. Federal District (FD), Brazil, 2014.

Regarding the absorbance of the reference test MAC-ELISA, the results were analysed in four groups divided by the intensity of the absorbance; 0.200 to 0.299; 0.300 to 0.399; 0.400 to 0.499 and ≥ 0.500. As expected, the rapid test IgM component obtained a better performance in samples with higher absorbance intensities, from 19% sensitivity for absorbance levels between 0.200 to 0.299 to 75% sensitivity for absorbance ≥ 0.500. The NS1 component sensitivity results again showed better performance than the IgM component (Fig. 3).

**Fig. 4:**
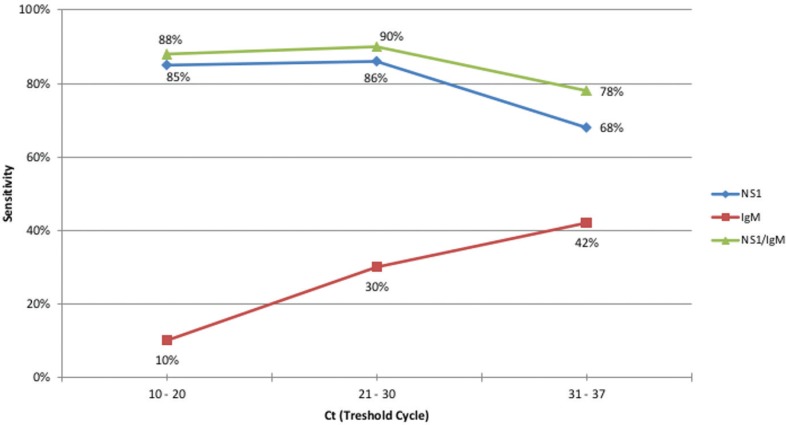
sensitivity evaluation of the NS1 component, IgM component and the combination of both NS1 and IgM components of the SD Bioeasy Dengue Duo test according to the Ct of the reference pattern real-time polymerase chain reaction (RT-qPCR). Federal District (FD), Brazil, 2014.

The sensitivity of the rapid test components in relation to the RT-qPCR reference test Ct was evaluated measuring Cts at intervals of 10 to 20, 21 to 30 and 31 to 37. The sensitivity of IgM increased with increasing Ct; the NS1 component had an inverse trend. We observed that the sensitivity of IgM ranged from 10% for Ct between 10 and 20 to 42% for Ct 31 to 37, while the NS1 showed a significant decrease from 86% for Ct from 21 to 30 to 68% for Ct 31 to 37 (Fig. 4).

The reliability study between health care settings with different levels of complexity was conducted for the IgM, NS1 and IgG components of the rapid test. We also analysed the agreement for samples classified as acute infection, that is, those positive samples for the NS1 and/or IgM. The best agreement was found for the NS1 component [Supplementary data (Table V)].

## DISCUSSION

The Brazilian experience with RDTs is still scarce, and there is national recognition that their introduction for clinical care or surveillance purposes should be thoroughly assessed, especially in relation to the effects that these tests will have on clinical, surveillance and disease control practices. Thus, it is imperative to have adequate accuracy and reliability evaluation before their incorporation for routine use.

In this context, the present research is the first phase III validation study of a rapid chromatographic immunoassay for the diagnosis of dengue conducted in Brazil. The dominant feature of the approach used was the prospective inclusion of over a thousand participants with suspected dengue clinical syndrome. Previous studies carried out in this country used samples stored in biorepositories or biobanks, affecting the performance of the index test largely due to the bias of previous serological screening. Another drawback of previous studies is the failure to comply with the manufacturer's instructions for rapid testing. Most of them require the test application in point-of-care settings, immediately after collection of biological samples, and not in frozen stored material (Boelaert et al. 2007).

Regarding the demographic and clinical characteristics of the participants, the average age was 35 years and there was a female predominance. Similar demographic profiles were observed in previous studies conducted in the state of Minas Gerais, Brazil, making the results of the present study comparable (Teixeira et al. 2010).

In this context, for this target population, the overall sensitivity of the SD Bioeasy Dengue Duo test was unsatisfactory. The best performance observed for the sensitivity was a moderate 76% for the combination of NS1 and IgM components. In other studies, conducted in different countries, the test sensitivity was higher at 85% (Andries et al. 2012) and 92% (Blacksell et al. 2011). This heterogeneity of available accuracy data reinforces the importance of evaluating the accuracy of RDTs in the target population where the test will be applied. This is relevant because of changes in genetic background, epidemiological and cultural conditions, among other factors that can directly affect the performance of RDTs. Specificity estimates of the SD Bioeasy Dengue Duo test performed well (98%), similar to other recent studies (Wang and Sekaran 2010, Blacksell et al. 2011).

The construction of an appropriate reference standard for validation was made possible by combining three reference methodologies. By comparing the three reference methodologies and the components of the rapid test, we were able to detect an unexpected pattern in the performance of the IgM component of the rapid test. Greater agreement was observed between positive results for the NS1 component than for the IgM component of the rapid test in samples with confirmed infection through the reference standard methodology MAC-ELISA IgM. This phenomenon may be associated with the lower sensitivity of the IgM component (35%), emphasizing the urgent need for improving this component.

Taking into consideration the overall accuracy in the present study, it can be suggested that the SD Bioeasy Dengue Duo test is useful to confirm but not enough to rule out the diagnosis of acute symptomatic dengue infection. This perception was confirmed by the positive likelihood ratio above 10 and negative likelihood ratio up to 0.1 (Grimes and Schulz 2005).

Currently, most rapid tests used in Brazil for the diagnosis of dengue are used in primary care settings as screening tests, despite their low sensitivity. Thus, the implementation of diagnostic tests without proper validation can bring new challenges to the health system. If rapid tests with unsatisfactory accuracy for the timely detection and management of cases were incorporated into health systems, we may have an increase in lethal outcomes.

The performance of the IgM and NS1 components together showed better results for acute compared to convalescent infection. In a study published in 2010 by Wang and Sekaran, similar data were observed. For the NS1 component, the sensitivity for acute and convalescent infection was 100% and 70%, respectively (Wang and Sekaran 2010). The lower sensitivity of the NS1 component of the test in relation to previous exposure to dengue infection may reflect the lower antigen levels common in people with reinfection (Bäck and Lundkvist 2013).

The analysis related to the period from symptoms onset to the time of sample collection demonstrated that the IgM component behaviour was consistent with the dynamics of infection by DENV. Our results demonstrated sensitivity that was directly proportional to the interval between the onset of symptoms and the collection of the sample. For the analysis combining NS1 and IgM results, there were no major variations in test sensitivity at any period of infection.

This study also provides data on the relationship between the magnitude of the antibody response and the positivity of the rapid test. The results showed that for NS1, IgM and IgM/NS1, components there was an improvement in test sensitivity when the concentration of IgM in the patient serum was greater. The results for the IgM test component were very consistent, demonstrating a direct relation to the intensity of the absorbance values.

We show, in this study, that the sensitivity of the rapid test components is directly related to the viral RNA concentration in positive samples. We observed a decrease in sensitivity of the NS1 component at a higher Ct. Lower Ct reflects a higher concentration of virus in the patient samples, and thus a higher concentration of viral antigens, such as the NS1 protein. These results demonstrate that the dynamics of patient infection directly affect the test results (Bäck and Lundkvist 2013).

We demonstrated a high reproducibility of the rapid test between the patient's point-of-care and reference laboratory results, reinforcing the potential usefulness of the test in the context of primary healthcare. The unsatisfactory results for the IgM component (Kappa = 0.5) and IgG component (Kappa = 0.75) may be expressing the influence of disease prevalence, reflecting the phenomenon known as Kappa paradox. When the prevalence rate is high, the correlation due to chance is high and, therefore, the Kappa will be lower (Sim and Wright 2005).

In conclusion, the rapid SD Bioeasy Dengue Duo test has high specificity and moderate sensitivity, and the values of the observed likelihood ratios suggest that the test is useful to confirm the diagnosis of dengue, but not enough to rule out such diagnosis and should be improved in respect to sensitivity. The combination of the two components, NS1 and IgM, had the best accuracy compared to the NS1 and IgM component performance when used separately. Our results are applicable to similar scenarios with transmission of DENV1 and should be applied cautiously in scenarios with significant circulation of DENV4, which has been reported as more challenging for achieving reasonable rapid test accuracy (Colombo et al. 2013, Acosta et al. 2014, Buonora et al. 2016). Regarding the dynamics of infection by DENV and immunological response, it may be concluded that patients who have triggered a greater immune response with higher concentrations of IgM and a higher viral load in blood are more likely to have positive results for the SD Bioeasy Dengue Duo rapid test. Reproducibility between observers of the selected health units and observers of the reference laboratory was satisfactory, reinforcing the test usefulness as a point-of-care test in the context of primary healthcare settings.
