# Supplementary material for: Validation and reliability of the rapid diagnostic test ‘SD Bioeasy Dengue Duo’ for dengue diagnosis in Brazil: a phase III study
Source: Mem Inst Oswaldo Cruz. 2018 Jun 25;113(8):e170433. doi: 10.1590/0074-02760170433 (PMC6014722; doi:10.1590/0074-02760170433)
Supplement: Supplementary file 1 [file 0074-0276-mioc-113-08-e170433-Suppl01.pdf]

TABLE I

False negative and true positive result distribution for the rapid test SD Bioeasy Dengue Duo among categories defined by the result composition from the reference standard tests (MAC-ELISA IgM, virus isolation, and real-time polymerase chain reaction (RT-qPCR), Federal District (FD), 2014

Reference standard (composition)	False negative n (%)	True positive n (%)
Mac IgM + / Isolation - / qPCR -	33 (67.34)	84 (40.77)
Mac IgM - / Isolation + / qPCR -	1 (2.04)	1(< 1)
Mac IgM - / Isolation - / qPCR +	4 (8.16)	14 (6.79)
Mac IgM + / Isolation + / qPCR -	0	0
Mac IgM + / Isolation - / qPCR +	5 (10.24)	50 (24.27)
Mac IgM - / Isolation + / qPCR +	6 (12.24)	48 (23.30)
Mac IgM + / Isolation + / qPCR +	0	9 (4.36)
Total	49 (100)	206 (100)

TABLE II

Comparison between the rapid test SD Bioeasy Dengue Duo and the standard reference methodologies [MAC-ELISA, real-time polymerase chain reaction (RT-qPCR, and virus isolation)], Federal District (FD), 2014

		Sample number with positive test (%)			
Positive categories	Number (n)	SD IgM	SD NS1	SD IgG	SD NS1/IgM
MAC-ELISA	143	72 (50.34)	90 (62.93)	50 (34.96)	105 (73.42)
RT-qPCR	121	30 (24.79)	101 (83.47)	16 (13.22)	106 (87.60)
Virus isolation	58	3 (5.17)	57 (87.93)	1 (1.72)	51 (87.93)

TABLE III

Reliability of the rapid test 'SD Bioeasy Dengue Duo' in both acute and convalescent infection scenario, Federal District (FD), 2014

		Sensitivity (%) [CI 95%]			Specificity (%) [CI 95%]		
Infection category	Number (n)	SD IgM	SD NS1	SD IgM/NS1	SD IgM	SD NS1	SD IgM/NS1
Acute (≤ 7 days)	362	34.73 (58/167) [28.1 to 39.8]	68.86 (115/167) [62.1to 73.8]	78.44 (131/167) [72.1 to 83.8]	99.48 (194/195) [97.6 to 100]	98.97 (193/195) [96 to 99.9]	98.46 (192/195) [96 to 99.9]
Convalescent (> 7 days)	47	50 (13/26) [32.2 to 67.6]	53.84 (14/26) [35.3 to 70.6]	57.69 (15/26) [39.3 to 74.6]	95.23 (20/21) [87.1 to 100]	100 (21/21) [21 to 100]	95.23 (20/21) [87.1 to 100]

CC BY

TABLE IV

Sensitivity of the rapid test 'SD Bioeasy Dengue Duo' for cases with or without history of dengue infection, Federal District (FD), 2014

		Sensitivity [CI 95%]			
History of dengue infection	Number (n)	SD IgM	SD IgG	SD NS1	SD NS1/IgM/IgG
No	148	37.83<br>(56/148)<br>[31.1 to 42.8]	24.32<br>(36/148)<br>[18.1 to 29.8]	70.27<br>(104/148)<br>[64.1 to 75.8]	77.70<br>(115/148)<br>[71.1 to 82.8]
Yes	21	38.09<br>(8/21)<br>[18.4 to 57.6]	33.33<br>(7/21)<br>[13.4 to 52.6]	38.09<br>(8/21)<br>[18.4 to 57.6]	66.66<br>(14/21)<br>[64 to 67.9]

TABLE V

Crude agreement, prevalence index, and Kappa value for each component of the rapid test 'SD Bioeasy Dengue Duo'. Federal District (FD), 2014

	Crude agreement (%)	Prevalence index	Kappa value ($CI_{95\%}$)
Component NS1	98 (395/401)	0.59	0.93 (0.89 to 0.96)
Component IgM	98 (395/401)	0.96	0.50 (0.15 to 0.84)
Component IgG	99 (398/401)	0.96	0.75 (0.50 to 0.99)
Acute infection NS1/IgM	98 (394/401)	0.57	0.94 (0.90 to 0.97)
